# Free-Ranging Frigates (*Fregata magnificens*) of the Southeast Coast of Brazil Harbor Extraintestinal Pathogenic *Escherichia coli* Resistant to Antimicrobials

**DOI:** 10.1371/journal.pone.0148624

**Published:** 2016-02-04

**Authors:** Juliana Yuri Saviolli, Marcos Paulo Vieira Cunha, Maria Flávia Lopes Guerra, Kinue Irino, José Luiz Catão-Dias, Vania Maria de Carvalho

**Affiliations:** 1 Department of Pathology - Faculdade de Medicina Veterinária, Universidade de São Paulo, São Paulo, SP, Brazil; 2 Enterobacteriacea Laboratory, Instituto Adolfo Lutz, São Paulo, SP, Brazil; Second University of Naples, ITALY

## Abstract

Seabirds may be responsible for the spread of pathogenic/resistant organisms over great distances, playing a relevant role within the context of the One World, One Health concept. Diarrheagenic *E*. *coli* strains, known as STEC (shiga toxin-producing *E*. *coli*), and the extraintestinal pathogenic *E*. *coli* (ExPEC and the subpathotype APEC), are among the *E*. *coli* pathotypes with zoonotic potential associated with the birds. In order to identify health threats carried by frigates and to evaluate the anthropic influence on the southern coast of Brazil, the aim of this work was to characterize *E*. *coli* isolated from free-ranging frigates in relation to virulence genotypes, serotypes, phylogenetic groups and antimicrobial resistance. Cloacal and choanal swabs were sampled from 38 *Fregata magnificens* from two oceanic islands and one rescue center. Forty-three *E*. *coli* strains were recovered from 33 out of the 38 birds (86.8%); 88.4% of strains showed some of the virulence genes (VGs) searched, 48.8% contained three or more VGs. None of the strains presented VGs related to EPEC/STEC. Some of the isolates showed virulence genotypes, phylogenetic groups and serotypes of classical human ExPEC or APEC (O2:H7, O1:H6, ONT:H7, O25:H4). Regarding antimicrobial susceptibility, 62.8% showed resistance, and 11.6% (5/43) were multidrug-resistant. The *E*. *coli* present in the intestines of the frigates may reflect the environmental human impact on southeast coast of Brazil; they may also represent an unexplored threat for seabird species, especially considering the overlap of pathogenic potential and antimicrobial resistance present in these strains.

## Introduction

Wild birds may be carriers or reservoirs of pathogenic organisms and, especially the migratory ones, may spread microorganisms from local people and animals to those living at great distances [1]. Epidemiological studies have reported the dissemination of pathogenic bacteria among birds and humans, in addition to the spread of antimicrobial resistance [1–4]. Human occupation of natural areas is an important factor in the emergence and direct or indirect spread of pathogens among humans and wildlife [5]. Anthropogenic environments may enable free-ranging avian species to contact with humans. Wild birds, in turn, may represent a source of infection for humans, as well as pathogens of human origin that can infect these animals. As biological indicators of environmental pollution, wild birds may play a relevant role within the context of the One World, One Health concept [1,4].

Brazil has approximately 8000 km of coastline and is considered the greatest inter-coastal and subtropical coast in the word. In addition, the coast has the greatest population density in Brazil (http://censo2010.ibge.gov.br/apps/atlas/), which results in wide anthropic action. More than 130 species of marine and coastal birds have been catalogued along the Brazilian coastline, most of which are migratory species from the northern hemisphere and extreme meridional region [6]. Frigates (*Fregata magnificens*) are island seabirds that feed on fish and use coastal and oceanic islands for breeding. They are in the order of Pelecaniformes, and *Fregata magnificens* is the only species of the genus *Fregata* that is found on the Brazilian coast. Frigates search for food on the surface of the sea water and interact directly with other species of migratory birds and coastal fisherman communities [7].

*Escherichia coli* are Gram-negative bacilli that colonize the intestines of mammals and avian species shortly after birth as commensal organisms; this property enables *E*. *coli* to be largely used as an indicator of ambiental contamination [2,5,8]. However, some *E*. *coli* strains are pathogenic due to the acquisition of virulence genes (VGs) [9] that allow them to cause intestinal and extraintestinal diseases. These strains are distinguished from commensal ones due to the presence and expression of different combinations of VGs, which encode meaningful virulence factors such as fimbriae, invasins, evasins, toxins, and siderophores [9].

*E*. *coli* is established more easily in hosts that live in proximity to man [10,11], and pathogenic and/or antibiotic-resistant *E*. *coli* strains have been reported in the wildlife microbiota and in environments that have undergone human impact. Consequently, the presence of these bacteria may predict anthropic influence [8,11]. Diarrheagenic strains known as STEC (shiga toxin-producing *E*. *coli*), and extraintestinal pathogenic *E*. *coli* (ExPEC), are among the *E*. *coli* pathotypes with zoonotic potential associated with the birds [12,13,14]. ExPEC includes the subpathotype avian pathogenic *E*. *coli* (APEC), a relevant disease agent for poultry that is also recognized as a potential human pathogen [13,15,16,17]. Diseases caused by ExPEC have a worldwide distribution and constitute a public health concern due to the potential medical and economic impact regarding human health, as well as the productivity losses in veterinary medicine [9,14]

Several factors contribute to environmental contamination by pathogenic/resistant *E*. *coli* strains. Human and animal fecal waste dumped into aquatic sources by domestic, agricultural and industrial sewage, for instance, represent a significant public health threat in water environments [18,19], and waterfowl, in turn, may contribute as a link in the chain of this One Health issue [4].

In order to search for health threats and to evaluate the anthropic influence, this study sought to characterize *E*. *coli* strains isolated from free-ranging frigates according to their virulence genotype, serotypes, phylogenetic groups and antimicrobial resistance.

## Materials and Methods

### Sampling sites, birds and ethical statements

Four expeditions to oceanic islands along the São Paulo state coast were carried out to collect biological samples consisting of one cloacal swab from each bird (in 21 frigates, choanal swabs were also collected- Table 1). Alcatrazes Archipelago (24°06´S– 45°41´W; 2009) is included in the DELTA area established by the Brazilian Navy for military exercises and is the site of three expeditions. This natural environment is considered the largest nursery of seabirds in southeastern Brazil and one of the most biodiverse island environments [7]. Only a small section of the archipelago is protected and included in the Ecological Station of Tupinambás (ESEC Tupinambás), but the 34 frigates were captured and sampled on the main island, which is not protected. One expedition took place on Castilho’s Island (25° 16'S—47° 57'W; 2009) where only two frigates were sampled. This island is part of the Ecological Station of Tupiniquins (ESEC Tupiniquins), which is characterized as a conservation area. Biological samples were also collected from two frigates rescued 20 days before and maintained in a center for the rescue and rehabilitation of marine animals (GREMAR—Resgate e Reabilitação de Animais Marinhos). These two birds presented no signs of illness and no history of antimicrobial drug administration.

**Table 1:** Description of Strains, Sampling Sites, and Birds.

Sampling site	No. Samples (anatomic site) [No. birds]	No. of strains selected (anatomic site) [No. birds]
**Alcatrazes (2008/9)**	38 (19 clo, 19 cho)^a^ [19]	21 (19 clo, 2 cho) [14]
**Alcatrazes (2010)**	15 (15 clo) [15]	15 (15 clo) [15]
**Castilhos (2008)**	4 (2 clo, 2 cho) [2]	4 (1 clo 3 cho) [2]
**GREMAR (2010)**	2 (2 clo) [2]	3 (3 clo) [2]
**Total**	59 (38 clo, 21 cho) [38]	43 (38 clo, 5 cho) [33]

^a^ clo = cloaca, cho = choana.

All of the free-ranging birds appeared to be healthy and were captured randomly and restrained in strict accordance with the protocols approved by the Research Ethics Committee (Permit Number: 1368/2008 Comitê de Ética no Uso de Animais/ Faculdade de Medicina Veterinária e Zootecnia/ Universidade de São Paulo). All activities were performed in full compliance with federal permits issued by the Brazilian Ministry of Environment (Alcatrazes: ICMBio—Instituto Chico Mendes de Biodiversidade, Permit Number 2997/1; Castilho’s Island and GREMAR: ICMBio—Instituto Chico Mendes de Biodiversidade, Permit Number 16553–1).

### Isolation and strains

The swabs were plated on MacConkey Agar (Difco^™^) and incubated at 37°C for 24 hours. The identification of the isolates was performed according to the routine techniques of identification, including the biochemical identification kit (Probac ^™^, São Paulo, Brazil).

Three to five isolates of *E*. *coli* from each site (cloca and choana) of each bird were stored in brain heart infusion medium (Difco^™^, New Jersey, USA) with 80% glycerol [Invitrogen; 1:1(v/v)] at -80°C for further analysis.

### Virulence genotype and phylogenetic group determination

Polymerase chain reaction (PCR) was used to amplify genes of interest after DNA boiling extraction. Multiplex PCR for the detection of minimal predictors of the avian pathogenic *E*. *coli* (APEC) genes *iroN*, *iss*, *ompT*, *hlyF*, and *iutA* was performed as previously described [20]. For the detection of ExPEC virulence-related genes (*papC*, *papEF*, *sfa*, *afa*, *cnf1*, *hlyA*, *iucD*, *fyuA*, *and iha*), single PCR reactions were performed following previously defined protocols [21,22]. The *fimH/malX/ibeA* and *traT/cvaC* genes were searched in the triplex and duplex PCR reactions, respectively [21].

Genes related to EPEC/STEC (*eae*, *stx1*, *and stx2*) were identified following protocols described elsewhere [23–25]. Determination of the major *E*. *coli* phylogenetic group (A, B1, B2 and D) was performed using Clermont’s multiplex PCR-based method [26]. Amplified DNA was separated by electrophoresis on a 1.5% agarose gel, stained with 0.5 μg/mL of ethidium bromide and photographed under UV light. A 100 bp DNA ladder was used as a molecular size marker (Invitrogen, São Paulo, Brazil).

### Antimicrobial susceptibility testing

Antimicrobial susceptibility was determined by disk diffusion test according to international standards [27]. The following antimicrobials (CEFAR^™^) were tested: 10 μg ampicillin (AMP), 30 μg cephalexin (CFE), 30 μg cefoxitin (CFO), 30 μg ceftiofur (CTF), 30 μg cefotaxime (CTX), 10 μg streptomycin (STR), 10 μg gentamycin (GEN), 30 μg neomycin (NEO), 30 μg amikacin (AMI), 30 μg tetracycline (TET), 30 μg chloramphenicol (CLO), 300 μg nitrofurantoin (NIT), 300 UI polymyxin B (POL), 5 μg ciprofloxacin (CIP), 10 μg norfloxacin (NOR), 5 μg enrofloxacin (ENO), and 25 μg sulfamethoxazole/trimethoprim (SXT). The breakpoints that were not defined in the veterinary documentation [28] were taken from CLSI M100-S23 [29]. A strain was considered resistant when it presented resistance or intermediate resistance to at least one of the drugs. A given strain was considered multidrug resistant (MDR) when demonstrating resistance to three or more antimicrobial classes [30].

### Serotyping

Strains were serotyped according to standard international procedures using the tube agglutination test and currently available O (O1- O181) and H (H1-H56) antisera in a reference center for *E*. *coli* serotyping (Instituto Adolfo Lutz, São Paulo, SP, Brazil).

### Statistical analyses

The significance of the results was established using Fisher’s exact test (two-tailed). The level for statistical significance was *p*<0.05.

## Results

*E*. *coli* strains were recovered from 33 out of the 38 birds (86.8%, Table 1). Three to five *E*. *coli* strains were isolated from a total of 34 swabs (31 swabs from cloaca and 3 from choana), once in one bird *E*. *coli* strains were isolated from both, cloaca and choana. Considering the 34 swab samples positive for *E*. *coli*, isolates obtained from 27 (79.4%) samples presented the same phenotype and genotype and were regarded as having the same clonal origin, therefore, only one of each similar strain was considered. In seven samples (6 from cloaca and 1 from choana), 16 strains with different patterns were detected and consequently considered as having separate origins. Thus, for the purposes of presenting the results, 43 strains will be taken into account (Table 1).

Table 2 shows the distribution of 43 *E*. *coli* strains according to the phylogenetic groups. Of the strains, 13/43 (30.2%), 12/43 (28.0%), 10/43 (23.2%) and 8/43 (18.6%) were allocated into phylogroups A, D, B2, and B1, respectively.

**Table 2:** Distribution of 43 *E*. *coli* Strains According to the Phylogroup and Associated Features.

Phylogroup	Virulence genes	Resistance	Serotype	Origin^c^
**A**	*fimH*, *cvaC*, *traT*, *fyuA*, *iroN*, *hlyF*, *ompT*, *iss*	TET	ONT:H-	A
	*fimH*, *fyuA*, *ibeA*, *iroN*, *hlyF*, *ompT*, *iss*	AMP	NP^b^	A
	*iroN*, *hlyF*, *ompT*, *iss*	AMP	NT	A
	*fimH*, *fyuA*	AMP, TET, NOR, CIP, ENO, SXT^a^	NP	RC
	*fimH*, *fyuA*	-	NP	A
	*fimH*, *fyuA*	CFE, TET, STR^a^	NP	A
	*fimH*	AMP, TET, NOR, CIP, ENO, SXT^a^	NP	RC
	*fimH*	AMP, NIT	NP	A
	*fimH*	AMP	NP	A
	-	AMP	NP	A
	-	AMP, TET	NP	A
	-	STR	NP	A
	-	-	NP	A
**B1**	*fimH*, *traT*, *iroN*, *hlyF*, *ompT*, *iss*	TET, NOR, CIP, ENO, SXT^a^	NP	RC
	*fimH*, *fyuA*, *ibeA*	STR	NP	A
	*fimH*, *fyuA*	AMP	NP	A
	*fimH*, *malX*	AMP, TET, CLO, STR, NOR, CIP, ENO, SXT^a^	O8:H23	A
	*fimH*, *traT*	-	O102:H10	A
	*fimH*, *traT*	-	O102:H10	A
	*fimH*	TET	0179:H21	A
	-	AMP	NP	A
**B2**	*fimH*, *cvaC*, *malX*, *traT*, *fyuA*, *ibeA*, *iroN*, *hlyF*, *ompT*, *iss*	-	O25:H4	A
	*fimH*, *cvaC*, *malX*, *traT*, *fyuA*, *ibeA*, *iroN*, *hlyF*, *ompT*, *iss*	-	O25:H4	A
	*fimH*, *papC*, *cvaC*, *malX*, *traT*, *iucD*, *fyuA*, *ibeA*, *iroN*, *hlyF*, *ompT*, *iss*, *iutA*	AMP, CFE	O2:H7	A
	*fimH*, *papC*, *cvaC*, *malX*, *traT*, *iucD*, *fyuA*, *iroN*, *hlyF*, *ompT*, *iss*, *iutA*	-	ONT:H7	A
	*fimH*, *malX*, *fyuA*, *ibeA*	TET	ONT:H7	A
	*fimH*, *malX*, *ibeA*	-	ONT:H7	A
	*fimH*, *malX*, *fyuA*, *iroN*, *hlyF*, *ompT*	STR	O13:H4	A
	*fimH*, *fyuA*, *iroN*	-	ONT:H18	A
	*fimH*, *malX*, *fyuA*, *iss*	-	ONT:H14	A
	*fimH*, *fyuA*, *ibeA*	CFE	NP	A
**D**	*fimH*, *malX*, *fyuA*, *iroN*, *hlyF*, *ompT*, *iss*, *iutA*	-	O1:H6	A
	*fimH*, *malX*, *traT*, *fyuA*, *iss*	-	O1:H6	C
	*fimH*, *malX*	TET	O1:H6	A
	*fimH*, *malX*, *fyuA*	NIT	O1:H6	C
	*fimH*, *iroN*, *hlyF*, *ompT*, *iss*, *iutA*	TET	O24:H-	A
	*fimH*, *cvaC*, *traT*	TET	049:H49	A
	*fimH*, *malX*, *fyuA*	AMP	NP	A
	*fimH*, *fyuA*	-	ONT:H23	C
	*fimH*, *fyuA*	-	ONT:H10	A
	*fimH*	-	O88:H1	C
	*fimH*	-	O73:H41	A
	*fimH*	TET, STR	O169:H-	A

^a^ Multidrug resistant

^b^ Not performed

^c^ A- Alcatrazes Archipelago; RC- Rehabilitation Center; C- Castilhos Island

Regarding the VGs searched, 38/43 (88.3%) of the strains presented between 1 and 13 VGs, with an average virulence score (VS) of 3.5 VGs among the strains (Table 3).

**Table 3:** Distribution of 43 *E*. *coli* Strains According to the Phylogenetic Group and Frequency of Virulence Genes.

Virulence gene^a^	Phylogenetic group *n* (%)					
	A 13 (30.2)	B1 8 (18.6)	B2 10 (23.2)	D 12 (28)	Total 43 (100)	*p*-value^b^
***fimH***	9 (69.2)	7 (87.5)	9 (90)	12 (100)	38 (88.3)	NS
***papC***	0	0	2 (20)	0	2 (4.6)	0.0498
***cvaC***	1 (7.6)	0	4 (40)	1 (8.3)	6 (13.9)	0.0196
***malX***	0	1 (12.5)	8 (80)	5 (41.6)	14 (32.5)	0.0007
***traT***	1 (7.6)	3 (37.5)	4 (40)	2 (16.6)	10 (23.2)	NS
***iucD***	0	0	2 (20)	0	2 (4.6)	0.0498
***fyuA***	5 (38.4)	2 (25)	9 (90)	5 (41.6)	22 (51.1)	0.0093
***ibeA***	1 (7.6)	1 (12.5)	6 (60)	0	8 (18.6)	0.0008
***iroN***^c^	3 (23)	1 (12.5)	6 (60)	2 (16.6)	12 (27.9)	0.0171
***hlyF***^c^	3 (23)	1 (12.5)	5 (50)	2 (16.6)	11 (24.3)	NS
***ompT***^c^	3 (23)	1 (12.5)	5 (50)	2 (16.6)	11 (24.3)	NS
***iss***^c^	3 (23)	1 (12.5)	6 (60)	2 (16.6)	12 (27.9)	0.0171
***iutA***^c^	0	0	2 (20)	2 (16.6)	4 (9.3)	NS
**VS**^d^	2.2	2.1	6.7	3	3.5	

^a^None of the strains were positive to *papEF*, *sfa*, *iha*, *hlyA*, *cnf1*, *eae*, *stx1*, and *stx2*.

^b^Indicates prevalence rates of the virulence genes among B2 versus non-B2 isolates. Fisher’s exact test *p*-values are shown only when *p* was <0.05. NS-non-significant.

^c^Genes considered minimal predictors of APEC (avian pathogenic *E*. *coli*) virulence.

^d^VS = virulence score. A virulence score was calculated for each isolate as the sum and division of all virulence factors

Five isolates contained none of the genes searched, and a strain of serotype O2:H7, phylogenetic group B2, demonstrated all 13 ExPEC VGs (Table 2). Of the genes, *fimH*, the gene encoding the type 1 fimbriae, was the most prevalent. This gene was also distributed in isolates of all phylogenetic groups and serotypes (Tables 2 and 3). The *fyuA* gene, a marker of yersinabactin production, was found in 51.1% (22/43) of the strains and was positively associated with phylogenetic group B2 (*p* = 0.0093), as it was present in 90.0% of the strains belonging to this phylogroup (Table 3).

With the exception of the *fimH*, *traT*, *hlyF*, *ompT*, and *iutA* genes, all other VGs found were positively associated with phylogenetic group B2 (p <0.05). The *malX* gene was identified in 8/10 and 5/12 of B2 and D strains, respectively and is closely related to the B2 strains (p < 0.0001). The genes *papC and iucD* were detected only in B2 strains. Isolates from the B2 phylogroup had a VS of 6.7, while the strains allocated to the other phylogenetic groups showed a VS between 2.1 and 3 (Table 3).

The combination of five genes regarded as minimal predictors of virulence for avian pathogenic *E*. *coli* (APEC, *iroN*, *hlyF*, *ompT*, *iss and iutA*) was present in *E*. *coli* recovered from four out 34 (11.7%) birds sampled in Alcatrazes (Table 2). Another six strains revealed a combination of four of these genes (*iroN*, *hlyF*, *ompT*, *iss*); five of these strains were from Alcatrazes’ frigates, whereas only one was recovered from a bird in rehabilitation. The combination of the five APEC genes was shown in the strains classified in the phylogenetic groups B2 and D, whereas the presence of four genes (*iroN*, *hlyF*, *ompT*, *iss*) was verified in three, two and one strain from the A, B2 and B1 phylogroups, respectively (Table 2).

None of the isolates were positive for the ExPEC-related genes *papEF*, *sfa*, *iha*, and *hlyA* or for the EPEC/STEC linked genes *eae*, *stx1*, and *stx2*.

Of the serotyped strains (n = 25), serogroup O1 was the most prevalent, with 16.0% (4/25) of isolates, all of which belonged to serotype O1:H6 and phylogenetic group D. Eight out of 25 (32.0%) of the strains had non-typeable O antigen, but three of them showed flagellar antigen H7. Serotypes O25:H4 and O102:H10 were found in 8.0% (2/25) each, and the strains serotyped as O25:H4, ONT:H7 and O2:H7 were associated with phylogenetic group B2 and most of the virulence markers (Table 2).

Sixty-three percent (27/43) of the isolates showed resistance to at least one antimicrobial, while 11.6% (5/43) of the strains were MDR (three strains from birds sampled in the rehabilitation center and two recovered from Alcatrazes free-ranging frigates), including strains of phylogroup B1, serotype O8:H23, which was resistant to antimicrobials of six different classes (Table 2).

The prevalence of isolates resistant to each antibiotic was as follows: ampicillin and tetracycline, 30.2% each (13/43); streptomycin, 13.9% (6/43); sulfamethoxazole/trimethoprim, enrofloxacin, ciprofloxacin, and norfloxacin, 9.3% (4/43) each; cefalexin, 7.0% (3/43); nitrofurantoin, 4.6% (2/43) and chloramphenicol, 2.3% (1/43). All strains were susceptible to cefoxitin, cefotaxime, ceftiofur, gentamycin and polymyxin B. Isolates classified in phylogenetic groups A and B1 showed higher resistance than strains from phylogroups B2 and D. The patterns of resistance are shown in Table 4.

**Table 4:** Resistance Patterns of 27 Resistant Strains Among the 43 *E*. *coli* Isolated from Frigates.

Phylogenetic group (N)	Resistance pattern	*N* (% of total)	*N (*%) of resistant strains, *N* (%) of non-resistant strains
**A (13)**	AMP, TET, NOR, CIP, ENO, SXT	2 (4.6)	11 (85), 2 (15)
	CFE, TET, STR	1 (2.3)	
	AMP, NIT	1 (2.3)	
	AMP, TET	1 (2.3)	
	AMP	4 (9.3)	
	TET	1 (2.3)	
	STR	1 (2.3)	
	Non-Resistant	2 (4.6)	
**B1 (8)**	AMP, TET, CLO, STR, NOR, CIP, ENO, SXT	1 (2.3)	6 (75), 2 (25)
	TET, NOR, CIP, ENO, SXT	1 (2.3)	
	AMP	2 (4.6)	
	TET	1 (2.3)	
	STR	1 (2.3)	
	Non-Resistant	2 (4.6)	
**B2 (10)**	AMP, CFE	1 (2.3)	4 (40), 6 (60)
	CFE	1 (2.3)	
	TET	1 (2.3)	
	STR	1 (2.3)	
	Non-Resistant	6 (14.0)	
**D (12)**	TET, STR	1 (2.3)	6 (50), 6 (50)
	AMP	1 (2.3)	
	TET	3 (7.0)	
	NIT	1 (2.3)	
	Non-Resistant	6 (14.0)	
**Total (43)**			27 (62.8), 16 (37.2)

## Discussion

Environmental pollution due to waste discharge into streams, rivers, estuaries and sea water is one of the factors favoring the dissemination and transfer of human and domestic animal pathogens to wildlife; the same factors also contribute to the spread of antimicrobial-resistant bacteria [5,12,19]. Moreover, bacteria present in wildlife’s microbiota may indirectly indicate anthropic influences and may represent an important wildlife health threat [4,5]. In this study, biological samples from free-ranging frigates living in natural sites and samples from newly caught frigates housed in a rehabilitation center were sampled for pathogenic and/or antimicrobial resistant *E*. *coli*.

*E*. *coli* strains were recovered from cloacal samples of most of the frigates and from the choanal samples of three birds. Gordon & Cowling [10] investigated the *E*. *coli* prevalence in vertebrates from Australia and concluded that among other factors, proximity to places inhabited by humans make avian species more likely to harbor *E*. *coli* because its prevalence in birds is usually low. To our knowledge, there are no extensive field studies on the microbiota profiles of free-ranging frigates, but it is noteworthy that *E*. *coli* strains were recovered in a high percentage of the birds inhabiting the southern coast of Brazil. In addition, 88.4% of the strains in this study showed some of the VG searched, 48.8% had three or more VG, and 62.8% were resistant to antimicrobials.

ExPEC, including specific pandemic lineages, are responsible for a significant incidence of disease processes in humans and domestic animals and have been linked to disease in wild animals kept in captivity [31], but ExPEC strains are rarely isolated from free-ranging wildlife [9,11]. Considering that the highest human population density in Brazil is concentrated close to the coastline (http://censo2010.ibge.gov.br/apps/atlas/), the results of the present work might reflect the wide human action that contributes to the spread of potential pathogens to wildlife, and some of the findings discussed above may support this hypothesis.

Studies related to the population genetics of commensal *E*. *coli* have verified that in animals, strains belonging to the B1 phylogenetic group are predominant (41%), whereas A is the major phylogroup present in the microbiota of humans (40.5%). On the other hand, in both, animals and humans, the percentage of B2 commensal strains present is approximately 20–25%, while D strains are represented by nearly 17% [11]. The strains present in the tested frigates were classified as belonging to the A phylogroup (30.2%), followed by D (28.0%), B2 (23.2%) and B1 (18.6%). The frigate-associated strains were 81.4% phylogroup A, which is linked to the human commensal strains, and B2 and D phylogenetic groups, which primarily contain ExPEC associated with humans and pets [9,32,33]. Further studies are necessary to determine whether these findings represent a shift in the frigate’s *E*. *coli* population distribution; however, *E*. *coli* genotypic characterization may denote a bird’s health threat as well as the pathogenic potential of these strains in humans.

It is interesting to highlight that studies performed in Brazil on broiler chickens with colisepticemia [34] and severe cellulitis [35] have shown that strains from phylogroup D and A, respectively, were the most represented, although strains classified into B2 had the greatest pathogenicity score. Furthermore, in another Brazilian study with diseased turkeys, APEC strains belonging to phylogroup B2 were the most prevalent. Likewise, these isolates showed a high virulence score rate compared with the other phylogenetic groups [36]. The results concerning the *E*. *coli* strains found in the frigates corroborate these finds. Although recovered from apparently healthy frigates, B2 strains had the highest number of searched ExPEC VGs (virulence score = 6.7), ten of which were positively associated with B2 phylogroup (*papC*, *cvaC*, *malX*, *iucD*, *fyuA*, *ibeA*, *iroN*, *hlyF*, *ompT* and *iss*). Some of the genes investigated were only positive in B2 strains (*papC* and *iucD*). These data are consistent with the literature that postulates that B2 ExPEC strains are potentially more virulent and, therefore, capable of causing more severe extraintestinal syndromes [21,37].

Avian colibacillosis is characterized as an extraintestinal infection [15], and molecular approach is one of the criteria used to characterize the pathogenic potential of avian *E*. *coli*. In this study, the presence of five plasmid-borne genes (*iutA*, *hlyF*, *iss*, *iron*, and *ompT*) considered minimal predictors for APEC virulence was assessed [20]. These genes are common to highly pathogenic APEC, and therefore, this genotype can distinguish APEC from avian fecal *E*. *coli* strains [20]. The combination of the five genes (*iroN*, *iss*, *hlyF*, *ompT*, *iutA*) was found in 9.3% (4/43) of strains, all from different free-ranging frigates from Alcatrazes. In addition, the combination of four genes (*iroN*, *iss*, *hlyF*, *ompT*) was detected in 13.9% (6/43) strains, two of which were recovered from the birds housed in rehabilitation. Both of these strains were multidrug resistant. Furthermore, grouped together, 23.3% (10/43) of the strain exhibited such predictor genes, and 40% (4/10) of them belonged to phylogroup B2. The former studies with APEC predictor genes have been performed with domestic avian species, but, although data on ExPEC pathogenicity for frigates is not available, the results obtained here should be of concern. Harboring strains with pathogenic potential may enable frigates to spread the bacteria among other waterfowl species sharing the same environment.

Some authors consider APEC an opportunistic pathogen, however, many recent studies have shown that particular APEC strains have very similar features to humans ExPEC isolates. Showing indistinguishable phenotypic and genotypic characteristics [15,13], these strains are capable of causing disease in mammals [38]. In our study, most of the strains exhibiting APEC predictor VGs were also equipped with a wide range of ExPEC virulence markers, including key genes associated with mucosal colonization, iron acquisition and bacterial invasion or evasion, in addition to markers of pathogenicity islands [9,21]. The association of these characteristics may indicate not only the pathogenic potential to the birds but also to humans, as discussed below.

The gene encoding type 1 fimbriae (*fimH*) was the most prevalent, with 88.3% positivity among the isolates. Although it is found in strains recovered from both healthy and ill individuals [14], this fimbriae has been demonstrated to have an important role in the initial colonization of tissues such as the tracheal epithelium of birds or the bladder epithelia of mammals. Thus, it facilitates the subsequent expression of other virulence factors [37].

Another relevant adhesin is P fimbriae, encoded by the *papC* gene, which was present in 4.6% (2/43) of the isolates. It is noteworthy that the *papC* gene in strains of avian origin is linked to the colonization of internal organs, but it is also an important adhesin among UPEC strains, especially those related to pyelonephritis [37]. One of the *papC*-positive strains was serotyped as O2:H7, while the other had an unidentified O antigen and an H7 antigen (ONT:H7). Both isolates were characterized in phylogenetic group B2 and showed a high virulence content (ONT:H7 strain exhibiting 12 VGs and O2:H7 13 of them, in addition to resistance to beta-lactams). The clonal group B2 O1/O2/O18:H7 is associated with human and pet extraintestinal infections, such as UTIs, pneumonia, osteomyelitis, and nosocomial infections [17,32,33]. The pandemic ST95 complex (*sequence type complex*) strains comprises these serotypes, which usually have antimicrobial resistance and are isolated from both diseased poultry and human neonatal meningitis, among other extraintestinal infections [33]. Riley (2014) reviewed ExPEC pandemicity and confirmed the necessity of improving our understanding of the epidemiology of this phenomenon, which is discussed taking into account primarily human and domestic animal isolates. Because wild birds can contact different environments and animal populations, the results obtained in the present work may provide insight into the role of wildlife in the spread of pathogenic *E*. *coli*.

Another relevant ExPEC VG is *malX*, a marker of pathogenicity islands (PAI 1_CFT073_) of the archetypal human ExPEC urosepsis strains [37]. This marker was verified in 32.6% (14/43) of frigates *E*. *coli* and was positively associated with the phylogroup B2. It was present in 8/10 (80.0%) strains classified in phylogenetic group B2, 5/12 (41.7%) strains of group D and 1/8 (12.5%) of group B1. It was always associated with other VGs. Comparing these results with those originating from poultry, the percentage of *malX*-positive isolates in frigates can be considered high because strains from birds with colisepticemia and the cloacal microbiota of healthy birds have showed lower *malX* frequencies [14,34,39]. Johnson et al. [17] compared ExPEC strains of human origin with APEC and found similar *malX* positivity in neonatal meningitis *E*. *coli* (NMEC) and UPEC strains but lower percentages in APEC. This finding was also verified by Maluta et al. [39] in studies of ExPEC strains of human and avian origin in Brazil.

*E*. *coli* invasion is facilitated by specialized systems for iron acquisition called siderophores that allow bacterial growth in environments with low concentrations of this metal. Three different systems were investigated in this work: the yersiniabactin system (encoded by the *fyuA* gene), aerobactin systems (*iucD* and *iutA*) and salmochelin (*iroN*). These genes were found in 51.1%, 9.3%, 4.6% and 27.9% of isolates, respectively, and the overlap of at least two genes occurred in 23.2% of the strains. The most prevalent, *fyuA*, is an important marker of virulence for UPEC, and it has also been associated with APEC pathogenicity [14]. This VG was present in isolates of serotypes O1:H6, O2:H7, and O25:H4, all of which were previously associated with pandemic ExPEC clonal lineages [33].

The ability to invade the bloodstream and brain endothelium has been linked to *E*. *coli* strains able to cause human extraintestinal infections. Among the virulence factors conferring this ability is an invasin encoded by *ibeA*, a gene closely associated with NMEC strains [9,13]. This VG is also significantly linked to poultry pathogenic strains but not to non-pathogenic strains [40]. In frigates, 18.6% of strains were positive for *ibeA*, a percentage close to that found by others studying APEC strains [14,40]. In Brazil, Cunha et al. (2014) [36] found an *ibeA* frequency of 31% in APEC strains from turkeys with respiratory disease, and this gene was positively associated with phylogroup B2. In the present work, six out of eight *ibeA*-positive isolates were classified in the B2 phylogroup, and most of *ibeA*-positive serotyped strains were O2:H7, ONT:H7 and O25:H4. According to Riley [33], the clonal group ST131 (sequence type 131), which has been reported worldwide, possesses the O25:H4 B2 serotype that is deemed a pandemic strain that emerged in the mid-2000s. Some of the prototypic ST131 VGs, such as *fimH*, *fyuA*, *traT*, *ompT* and *malX*, have been demonstrated in two B2 strains serotyped as O25:H4 that were isolated from different birds from Alcatrazes. This usually high virulence lineage is associated with urinary tract infection, septicemia, nosocomial and community-acquired infections [33], which reinforces the possible role of wild birds in the dissemination of pandemic strains.

Also regarding invasive strains, the outer membrane lipoprotein Trat, encoded by the *traT* gene, has been shown to be of great importance because it increases bacterial resistance to the lytic action of complement [37]. In this study, the *traT* gene had 23.2% frequency among isolates and was found in a wide variety of serotypes (O25:H4 O2:H7, O49:H49, O102:H10, and O1:H6). APEC of poultry origin tested by others [14,17,34,39] showed higher *traT* gene positivity. Rodriguez-Siek et al. (2005) [14] noted that among APEC, the presence of *traT* was associated with the *cvaC* gene. The *cvaC* gene is found primarily in extraintestinal virulent strains causing disease in humans and animals [14,17] and showed a prevalence of 13.9% in our study, always accompanied by *traT*. Both genes are part of the resistance mechanism to the bactericidal effects of serum and are generally associated with sepsis in birds [14,17].

It is interesting to highlight that, in addition to the presence of important virulence markers, the occurrence of serotypes associated with human and avian disease reinforces the hypothesis that wild birds may play a role in the epidemiology of ExPEC, even as they may also have their health threatened. Of the 12 serogroups identified in our study, six of them (O1, O2, O8, O25, O88 and O102) are predominant among APEC serogroups [14] and are involved in human community-onset and healthcare-associated infections, in addition to pet disease [32,33,39].

In contrast to the absence of knowledge regarding the role of wild birds in the epidemiology of ExPEC infections, there is some information on the involvement of wild birds in the dissemination of *E*. *coli* strains that are resistant to antimicrobials [2,3,4,41], and because of this, wild birds have been regarded as biological indicators of anthropic action [4].

In the current investigation, 62.7% (27/43) of the strains showed resistance to at least one antibiotic, and 11.6% (5/43) were multidrug-resistant. Ampicillin, tetracycline and streptomycin were the antibiotics with the highest number of resistant strains, similar to what has been reported by other authors [4,42]. In spite of the lower percentage, resistance to other antimicrobials, including first generation cephalosporins, fluoroquinolones, sulfonamide, chloramphenicol and nitrofurantoin, was also reported. These drugs have wide use in humans, pets and production animals, leading to environmental contamination [43] and the introduction of these antibiotics into the aquatic ecosystem [18,41], which favors the acquisition of bacterial strains with antibiotic resistance by wild birds that have not come into contact with antimicrobials.

It is interesting to note that although the B2 strains have a higher VG content, they showed a lower resistance profile, in contrast with A and B1 in which most strains appeared resistant. The phenomenon of a negative association between resistance and B2 background has been previously reported and may be related to the fact that resistant strains may originate from less virulent populations [44].

A point that should be highlighted is that the three strains recovered from frigates in the rehabilitation center were multidrug resistant, even though these birds had not been treated with antimicrobials. This issue should be considered in the rehabilitation and reintroduction of wild species. Wild animals may be colonized by resistant and/or potentially pathogenic bacteria in rehabilitation centers, and these bacteria may then be transmitted and disseminated to free-ranging populations in different geographical locations [45].

Finally, from the standpoint of the One Health approach, the presence of *E*. *coli* in the intestinal contents of frigates may reflect the environmental impact of humans along the southeast coast of Brazil. ExPEC may also represent a threat to seabird species and may contribute to the epidemiology of human ExPEC. Special attention must be paid to the reintroduction of rescued wildlife because maintenance in healthcare settings enables the selection of resistant bacteria, even in individuals not subjected to antimicrobial treatments, and this phenomenon may contribute to the spread of resistance genes in the environment. This is a reason for concern, especially considering the overlap of pathogenic potential in the strains identified in this study.
